# Dual-mark-guided entry technique for preventing entry-site deviation during peroral endoscopic myotomy

**DOI:** 10.1055/a-2871-7609

**Published:** 2026-05-22

**Authors:** Satoshi Abiko, Kei Ushikubo, Kazuki Yamamoto, Yohei Nishikawa, Ippei Tanaka, Haruhiro Inoue, Naoya Sakamoto

**Affiliations:** 1 Digestive Disease Center 378609 Showa Medical University Koto Toyosu HospitalKotoTokyoJapan; 2 Department of Gastroenterology and HepatologyHokkaido University HospitalSapporoJapan

In peroral endoscopic myotomy (POEM), accurate identification of the 2 o’clock position of the lower esophageal sphincter (LES), located between the anterior and posterior sling fibers, is crucial for minimizing postoperative gastroesophageal reflux and achieving durable outcomes 1 2 3 . Initiating mucosal incision at the 2 o’clock position on the esophageal side enables smooth progression toward the corresponding LES position and facilitates formation of a linear submucosal tunnel extending to the gastric lesser curvature; however, unintended entry-site deviation may occur due to loss of accurate orientation in direction and height, particularly among novice operators. Therefore, we developed a technique in which two reference marks are placed at the intended entry site and connected by a mucosal incision (dual-mark-guided entry technique).

**Fig. 1:**
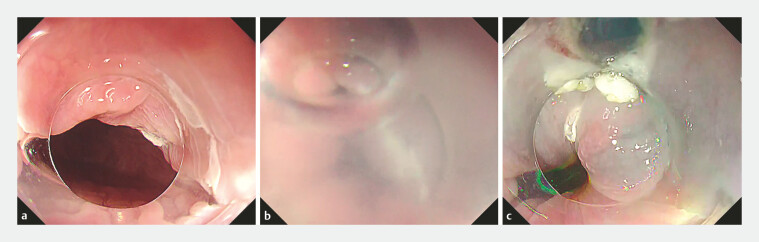
Images showing use of the dual-mark-guided entry technique for peroral endoscopic myotomy entry. **a** The esophageal level and 2 o’clock orientation were identified using the position of the left main bronchus, water surface level, and insertion depth of the endoscope and two marks were placed at the intended entry site. **b** Submucosal injection was initiated from the oral-side mark, after which, a positional shift toward the 3 o’clock direction was observed and confirmed using the EndoBubbloMeter, an orientation-assessment tool that visualizes endoscope direction by monitoring air bubbles within the distal attachment cap. **c** To maintain the intended height and orientation, the mucosal incision was created by connecting the oral- and anal-side marks.

**Fig. 2:**
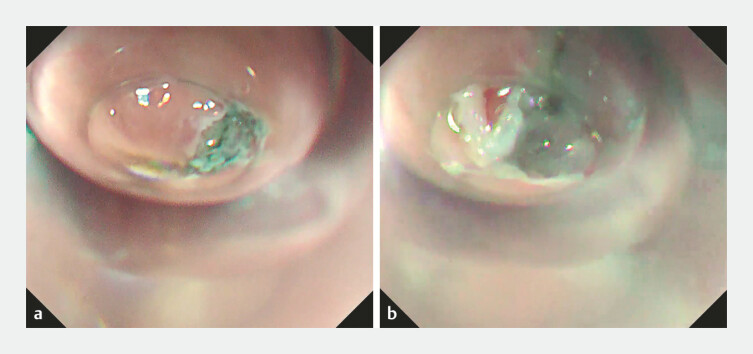
Images showing the EndoBubbloMeter confirmed that the mucosal entry was located at the 2 o’clock position of the esophagus after completion of the incision. **a** Mucosal incision at the oral-side. **b** Mucosal incision at the anal-side.

The dual-mark-guided entry technique was used for POEM entry. The esophageal level and 2 o’clock orientation were identified using the position of the left main bronchus, water surface level, and insertion depth of the endoscope and two marks were placed at the intended entry site ( Fig. 1 **a** ). Submucosal injection was initiated from the oral-side mark, after which a positional shift toward the 3 o’clock direction was observed and confirmed using the EndoBubbloMeter, an orientation-assessment tool that visualizes endoscope direction by monitoring air bubbles within the distal attachment cap 4 ( Fig. 1 **b** ). If the mucosa were incised at the presumed 2 o’clock position after injection, the incision could be displaced leftward. Therefore, to maintain the intended height and orientation, mucosal incision was created by connecting the oral- and anal-side marks ( Fig. 1 **c** ). After completion of the incision, the EndoBubbloMeter confirmed that the mucosal entry was located at the 2 o’clock position of the esophagus ( Fig. 2 and Video 1 ).

Use of the dual-mark-guided entry technique for peroral endoscopic myotomy entry.Video 1

The dual-mark-guided entry technique may ensure accurate mucosal entry during POEM.
